# Opening the Door Wider to Community Support of People With Serious Mental Illnesses: What States Can Learn From the IDD Experience

**DOI:** 10.1111/1468-0009.70110

**Published:** 2026-07-29

**Authors:** HAROLD POLLACK, RICHARD FRANK, SHERRY GLIED

**Affiliations:** ^1^ Crown Family School of Social Work Policy, and Practice University of Chicago; ^2^ Brookings Institute; ^3^ NYU Wagner Graduate School of Public Service

**Keywords:** Serious and persistent mental illness (SPMI), intellectual and developmental disabilities (IDDs), deinstitutionalization, Medicaid 1915(c) waivers, Medicaid 1115(b) waivers

## Abstract

**Policy Points**

- The federal government should provide states with authorities under Medicaid that allow greater use of home and community‐based services for people with serious and persistent mental illness.
- This requires deemphasizing authorities that require budget neutrality in a post‐institutionalization world (Section 1115 waivers) and relying on those with greater budget flexibility (Section 1915 waivers).
- The federal government, in its design of new mental health programming, would make it most effective if it establishes regulations and oversight that permit states to effectively pursue policy intents.
- When federal programs that intend to deal with consequences of serious and persistent mental illness are provided through broad authorities (e.g., 988 call lines), states should take greater care to create expectations and lines of accountability that align with federal intent and limit tendencies toward mission drift.

**Context:**

Since the early 1960s, US policymakers have sought to assist persons living with intellectual and developmental disabilities (IDDs) and persons living with severe and persistent mental illness (SPMI) to live on a human scale with their clinical and service needs met in their communities.

**Methods:**

We review this history, including the accomplishments and shortcomings of these efforts.

**Findings:**

People with both sets of disabilities and their families have benefited from deinstitutionalization. Yet deinstitutionalization proved tangibly more successful in assisting people with IDDs and their families than have comparable efforts that sought to assist people with SPMI and their families.

**Conclusions:**

These contrasting histories offer instructive lessons for state policymakers and others seeking to improve services for people living with SPMI.

Over the past six decades, the focus of federal and state policy addressing people living with intellectual and developmental disabilities (IDDs) and those living with severe and persistent mental illness (SPMI) has shifted from financing institutional care to providing a range of services in the community. Yet the experience of these two groups through the period following deinstitutionalization has been quite different. While many people with SPMI are better off today than they would have been in the era before deinstitutionalization, a substantial minority of the estimated 14.6 million Americans experiencing SPMI (about 5.5% of the adult population) live in very poor conditions.^1^, ^2^ Far too many are homeless,^3^, ^4^, ^5^ and violent encounters between police officers and people with SPMI remain a serious challenge.^6^, ^7^, ^8^, ^9^ By contrast, although people with IDDs continue to face many challenges, policy directed at this group has made tangibly greater progress in promoting greater autonomy and self‐direction, greater community integration, and higher quality of life,^10^ with less evidence of leaving significant subpopulations in terrible circumstances. Understanding why these experiences diverged, and how the design and implementation of policy have contributed to this divergence, can inform the design of better policies to serve people living with SPMI.

This comparison is particularly informative because the populations share many similarities in addition to their need for long‐term supports and history of institutionalization. An estimated 2.99 million children and 3.03 million adults live with cerebral palsy, intellectual disabilities, or autism spectrum disorders, and roughly 40% as many as live with SPMI. An estimated 608,000 persons age 21 or younger are known or served by state IDD agencies, alongside an estimated one million adults age 22 or older.^11^

The IDD and SPMI populations each account for significant shares of Medicaid spending. There are no estimates of spending on the SPMI subpopulation, but Medicaid spending on all mental health care in 2019 has been estimated to range from $58 billion (about one‐third of projected total mental health spending in that year) to $82.5 billion (or 49.6% of total mental health spending).^12^, ^13^ Total Medicaid spending on the IDD population in 2022/23 was $86.9 billion; other public sources spent a further $17.6 billion on behalf of this population. ^89^

The two, sometimes‐overlapping populations share history, governance, and program infrastructure. In both cases, states have historically been responsible for providing services. Until the mid‐1960s, states discharged that responsibility by relying heavily on institutionalization of people with intense service needs, overseen by similar state bureaucracies for both populations. States provided minimal community‐based services for either population or for family caregivers.

Both populations experienced rapid deinstitutionalization during the 1960s and 1970s. As late as 1977, the US Government Accounting Office (GAO) grouped the two populations together under the umbrella term “Mentally Disabled” in an analysis of the post‐deinstitutionalization landscape.^14^ The GAO pointed out the following:

When persons are patients or residents in public institutions, responsibility for their care is usually evident…When mentally disabled persons are released from institutions, however, responsibility for their care and support frequently becomes diffused among several agencies and levels of government…Deinstitutionalization has not received the full and well‐coordinated support of many State and local agencies administering programs that serve or can serve the mentally disabled.

Implementing policy at the state level has continued to be a challenge. States vary considerably in how they organize, finance, and manage their programs serving people with IDDs and SPMI. Some centralize governance. Others outsource most care provision to counties and localities. Some channel Medicaid behavioral health and IDD funding through general managed care organizations. Others carve it out to specialized managed care organizations (particularly for behavioral health). Still others continue to use fee‐for‐service financing. Yet the divergence in outcomes between SPMI and IDD is widespread and does not appear to be systematically related to organization of state governance. For the provision of care for people with SPMI, despite decades of arguments over carve‐ins and carve‐outs, the evidence base supporting any of these structures remains mixed and generally inconclusive.^15^

At the federal level, policy affecting the IDD population has largely been embedded within Medicaid and disability policy since the 1970s, particularly through the continued expansion of and emphasis on home and community‐based services (HCBS). Policy focused on people with SPMI has varied more across administrations. Recent Democratic administrations have emphasized coverage expansion, support for nonpolice crisis response, measures to safeguard the legal rights of people who live with these conditions, and the creation of mental health and drug courts and other diversion or deflection interventions.^2^, ^5^, ^16^, ^17^, ^18^, ^19^, ^20^, ^21^ The Trump administration, by contrast, has been promoting civil commitment and institutionalization of people with SPMI. These are significant and consequential differences. To date, however, they have not translated into a substantial narrowing or broadening in the difference between the experience of the IDD and SPMI populations (though recent Medicaid changes that may particularly affect those with SPMI could alter this conclusion).

In this article, we argue instead that the challenges of state implementation today stem from policy decisions made during the period of deinstitutionalization that persist to the present. We shed some light on the disappointing outcomes of SPMI policy by comparing the history and circumstances of those with SPMI with those for the IDD population, a group of people with some overlapping needs, for whom policy intentions have been more successfully put into practice. We argue that an important factor in the shortfall in the experience of people with SPMI is that policy for SPMI, in contrast to that for IDD, has not produced a unified, coherent, and accountable system of care, leaving services fragmented across programs and insufficient to meet the full range of needs required for community living.

Our article proceeds as follows. In the first three sections, we review the shared history of institutionalization and deinstitutionalization for people with SPMI and IDD. We then show how subsequent policy—especially within Medicaid—created a more coherent system of financing and support for IDD than for SPMI. Next, we demonstrate how these differences gave rise to the fragmented system of care that now serves people with SPMI. In the fourth section, we use recent policy initiatives to illustrate how this fragmentation shapes implementation and outcomes. We conclude with implications for strengthening community‐based care for people with serious mental illness.

## The History and Legacy of Deinstitutionalization

We begin with the shared history of institutionalization and deinstitutionalization. During the period of institutional care, the experiences of people with SPMI and IDDs had many similarities. The policy choices that followed deinstitutionalization, however, produced very different systems of financing, accountability, and community support.

### Institutionalization and State Responsibility

Beginning in the 19th century, states and counties have held responsibility for addressing both SPMI and IDD populations. For much of that history, states and counties largely exercised those responsibilities by building and operating institutions. In 1955, at the height of mental health institutionalization, the daily census of state and county mental hospitals^1^ averaged 559,000 people. These institutions accounted for an estimated 57% of total mental health spending in the United States as late as 1972.^22^ In 1965, at the height of institutionalization of persons living with IDDs, more than 190,000 people were housed in similar state and county institutions.^23^, ^24^

In parallel with these physical institutions, states established bureaucracies and governance structures to oversee mental health services’ financing and operations.^14^ Their arrangements varied across states. In most states, governance typically involved a dedicated state office that set policy and regulations, dealt with workforce policy, and provided institutions with their operating budgets. State or county offices oversaw the operations of these institutions.^25^, ^26^, ^27^ The balance of responsibilities between states and local/county/regional authorities differed among states, but states with more centralized state governance of SPMI services often (but not always) implemented centralized governance of IDD services.^28^

By the time these institutions reached their peak populations in the 1950s and 1960s, conditions in state and county institutions were generally poor and were the sources of scandalous treatment of residents that included forced labor, harmful medical practices (e.g., lobotomies), and other abuses that included the continual confinement of persons who did not require intensive levels of care.^29^ Although accountability was fully vested in states, most did a poor job of monitoring conditions or ensuring that persons confined to institutions were best served in those settings. Ronald Conley (1973) captured the urgency of public reactions to the poor conditions of institutional care: “States are eagerly seeking methods of providing alternatives to institutional care. This movement appears to be gaining momentum concurrently with growing public revulsion at the intolerable standards of care existing in most public institutions.”^30(p. xiii)^

Even at the height of institutionalization, only a minority of people living with SPMI or with IDDs were institutionalized—about 7%‐12% of those with SPMI^31^ and between 3% and 9% of the population with IDDs, depending on the definition used.^23^Many people with these conditions lived with their families or independently. Nonetheless, through the mid‐1960s, near‐universal pejorative assumptions regarding the functional capacities of persons with IDDs or SPMI, combined with the lack of educational pathways or home and community services to meet the needs of people with these conditions and their loved ones, led many families to see institutional care as the only viable option, particularly when a loved one experienced (or was viewed as likely to experience) severe symptoms related to these conditions.^29^ Popular descriptions, such as Pearl Buck's account^32^ in her 1950 bestseller *The Child Who Never Grew* of her decision to institutionalize her nine‐year‐old daughter Carolyn, captured the experiences and accompanying mindset of many parents and caregivers supporting loved ones with intellectual disabilities. Explaining her decision to institutionalize Carolyn, age nine, at the Vineland Training School, Buck writes: “I realized then that I must find another world for my child, one where she would not be despised and rejected, one where she could find her own level and have friends and affection.”

As recounted in her daughter Janice Walsh's poignant afterword to *The Child Who Never Grew*, Pearl Buck overstated the severity of Carolyn Buck's disability. Carolyn was an avid athlete, who enjoyed cooking and other hobbies. Given proper supports by year 2026 standards, she was well equipped to live on a human scale in the broader community. James Trent, in his authoritative history *Inventing the Feeble Mind*,^29^ observed that many parent memoirs of the times recounted similar incidents, in which professionals bluntly recommended institutionalization as the only possible course. A 1955 story, “Unfit Mother,” specifically rebuked a mother who refused to institutionalize her disabled son, thereby neglecting her husband and other children.

For decades, service providers were often expected to ease parents’ guilt and pain by emphasizing the necessity and inevitability of institutional care. The first author of this article is co‐guardian of a 60‐year‐old man who lives with Fragile X syndrome and moderate accompanying intellectual disabilities. During the late 1960s, medical providers repeatedly urged his parents to commit him to institutional care, citing the intensity of caregiver burdens they were likely to assume.^33^ Family members of persons living with SPMI often encountered similar recommendations for institutionalization, facing equal or greater caregiver burdens and substantially less capable systems of community supports to meet their loved one's needs outside institutional walls.

### Deinstitutionalization and Divergent Policy Paths

The fact of deinstitutionalization alone does not explain today's differences between the two populations. Rather, the financing and governance structures that replaced institutions evolved in very different ways for people with SPMI and people with IDDs.

*The Transition to Community‐Based Care*. The post‐World War II period ushered in an era of therapeutic optimism with respect to SPMI. The experience of psychologists and psychiatrists successfully treating mental illnesses related to combat, the discovery of new antipsychotic and antidepressant medications, and the rise of psychoanalytic practices led to a view that most mental illness could be successfully managed in community settings. Institutionalization was increasingly viewed as a last resort, reserved for those without adequate support, those with behaviors that were deemed dangerous to themselves or others or highly disruptive, or those with severe conditions that placed untenable burdens on these persons and on their caregivers, given the immediately available community resources.

This change in attitudes was complemented by a change in funding patterns that affected both populations. Until 1965, the federal government accounted for 9%‐12% of spending on health care. States bore virtually the entire burden for long‐term services and supports for people living with SPMI or IDDs. As described by Braddock, “the Federal presence in intellectual disability was previously so modest that a grant of $1.25 million from the Kennedy Foundation in 1952 to establish a private school in Illinois exceeded the entire Federal services budget for intellectual disability at that time.”^34^

These patterns began to change with the introduction of the community mental health centers (CMHC) program in 1963 and Medicaid in 1965, though as late as 1969, the federal government spent only $201 million on direct services to persons with IDDs.^35^

Medicaid provided a federal match for state expenditures on medical care, but it excluded coverage for care provided to adults under age 65 in institutions for mental diseases.^36^ This incentive, along with changes in psychiatric practice and social norms around individual rights, and, eventually, a series of legal cases,^2^ led states to move people out of traditional institutions and dramatically reduce institutions’ number and size. Associated expenditures, particularly to serve persons with IDDs, began to accelerate during the Nixon and Ford administrations.^35^ The Supplemental Security Income (SSI) program, which began in 1972 (Public Law 92‐603) also facilitated deinstitutionalization by providing a steady, albeit modest source of income to people who were adjudicated as unable to work because of a disability.^35^, ^37^

The introduction of these programs produced rapid change for the institutionalized populations with SPMI. In 1955, about 75% of mental health spending went to cover expenses for the roughly 559,000 people residing in public mental hospitals.^22^ By 1975, the number in mental hospitals had fallen to 212,000 people, and by 2014, to 101,000 people residing in various mental hospitals (e.g., state hospitals, VA, private psychiatric facilities). Today it is estimated that 20%‐30% of all US spending on mental health care is for institutional care. By January 1, 2025, an estimated 36,542 patients were housed in state psychiatric hospitals, more than a 90% reduction in patient population since the 1950s. Current annual expenditures for state psychiatric hospitals now total approximately $14 billion, roughly 25% of state mental health agency spending, which is less than one‐third of the $44.6 billion in state mental health agencies’ annual expenditures for community mental health care.^38^, ^39^

Reformers understood from the outset that the new programs alone were inadequate for many of those who were or would have been in institutions. Institutions had provided people with housing and food, basic caregiving, psychiatric services, and some medical care, and as a consequence of their design had effectively coordinated these services. These institutions had also separated people who might engage in dangerous, disruptive, or self‐injurious behaviors from vulnerable family members and their home communities—though they did not always separate people who might exhibit such behaviors from other vulnerable residents in the same institutions of care. From the start, advocates for deinstitutionalization recognized the need for more comprehensive services outside the institution. President John F. Kennedy championed and ultimately signed the Community Mental Health Act in 1963 that established CMHCs that were intended to provide comprehensive mental health and medical care to people with serious mental illnesses, especially those reentering community life from state and county mental hospitals. This vision was never fully realized. Swanson and colleagues summarized the accompanying consensus among researchers, clinicians, policymakers, and affected stakeholders^40^: “The widespread closure of US psychiatric hospitals and general psychiatry units over a period of decades without a reliable community care system to replace them has been recognized as a policy failure contributing to lack of treatment and poor outcomes for adults with disabling psychiatric conditions.”

The failings noted by Swanson and colleagues are tied to a set of policy choices at both the federal and state levels. The historical state (and county) governance structures developed little accountability for and provided few services to those outside institutions. That was in part because policies governing the creation of CMHCs largely bypassed established state mental health systems.^23^, ^41^ Medicaid, too, dramatically changed the role of state government vis‐à‐vis the public mental health system. Rather than establishing budgets and directly overseeing care delivery, the state increasingly took on the role of insurer. The result was that community mental health grants entirely bypassed state mental health agencies. Medicaid paid providers directly for their services, and the state set some aspects of the benefit design and prices to providers.^23^ ^,41^That meant that the state agencies that had explicit responsibility for people with mental illnesses were decoupled from the bulk of new funding for mental health care, experienced budget reductions, and were excluded from governance of new organizations (CMHCs) that resulted from federal grant funding initiatives. As a result, the organizations charged to administer the new funding streams had little expertise to exercise oversight at the state and local levels.

The new programs, Medicaid and the CMHCs, also came up short for several reasons.

First, these new institutions and systems were challenged in their ability to specifically address the needs of people with SPMI who might otherwise have been institutionalized. Because CMHCs were entirely federally funded and organized, they were disconnected from state government. Individual CMHCs were given great leeway to “serve their communities.” Too often, care and support of people with SPMI was deemphasized in favor of addressing the real, but less serious, mental health and social problems of other community members.

Second, many of the services needed by people who were at risk of institutionalization—foremost among them housing, but also supports for family members facing a loved one's risks of disruptive, self‐injurious, or dangerous behaviors—were outside the scope of state and county mental health offices and have generally been poorly funded.^42^ Modern housing supports for people with SPMI depend on diverse financing streams and regulatory policies at the state level. There is also substantial variation in the willingness and ability of states to complement the limited funding from the Department of Housing and Urban Development (HUD) with state and local funds to create affordable supported housing projects. Finally, varied state and local approaches to zoning, alongside efforts by local communities that commonly exclude stigmatized services and populations, limit what is possible in creating affordable supported housing projects.

Third, the availability of federal matching funds for Medicaid led states to prioritize services that qualify for the federal match, which are largely clinical services for people with SPMI.

Fourth, while evidence‐informed treatments for people with SPMI are effective, they have not fully achieved the hopes of reformers. It has proven challenging to organize routine treatment delivery to ensure that care will realize results that the evidence promises based on model programs and interventions. For many people, treatment that fully adheres to guidelines alleviates some symptoms but does not restore full functioning. For example, psychosocial support programs such as supportive employment improve the well‐being of people with SPMI but rarely result in earnings that make participants economically self‐sufficient.

Finally, many of the new organizations and service programs for people with SPMI were and continue to be funded through federal grants to states. Yet these grant mechanisms establish program rules that states must accept, and they can contain onerous provisions poorly aligned with state delivery system circumstances. For example, in the recent effort to promote Certified Community Behavioral Health Clinics (CCBHCs), certification requirements imposed scales of operations and costs that proved infeasible for many low‐income, sparsely populated, or rural communities. In other cases, grants to the states included few accountability requirements regarding service quality and coordination. Together this created a complex array of programs with fragmented governance that sometimes impeded the purpose of expanding availability of services.^43^

*Medicaid HCBS and the Emergence of a Coherent IDD System*. The decline in IDD institutionalization took off much later than the corresponding decline for SPMI.^44^, ^45^ At the 1967 peak, public residential facilities (PRFs) that served 16 or more people had an average daily population of 194,650, with another 33,850 Americans with IDDs living in state psychiatric facilities.^24^ As recently as 1980, 131,345 Americans with IDDs lived in PRFs, with another 9,405 living in state psychiatric facilities. By 2021, these number had declined by 89% (see Larson and colleagues Table 4.10).^46^

The population composition of IDD institutional care had also changed, to serve an older, more complex, and more costly population that often exhibits challenging behaviors or experiences other intense needs. In 1977, fully 36% of PRF residents were age 21 or younger, whereas 23% were over age 40. By 2021, only 4% of PRF residents were age 21 or younger. Seventy‐three percent were over age 40 (see Larson and colleagues [2025] Table 4.14).^24^, ^46^ An estimated 66% of 2021 PRF residents were indicated to live with severe or profound intellectual disabilities; 60% had indicated behavioral disorders requiring planned intervention. A striking 23% of new PRF admissions in calendar year had a correctional facility indicated as their previous place of residence. In 1985, 39% of new admissions had an indicated previous place of residence as the home of parents or other relatives. By 2021, this proportion had dropped by more than two‐thirds: Only 12% had an indicated previous place of residence as the home of parents or other relatives. Such changing patterns are consistent with expectations that community‐situated residential settings offer markedly less advantage over institutions in addressing challenging behaviors.

In part, deinstitutionalization of the IDD population was slower because many of those institutionalized with IDDs were under age 21, and federal Medicaid rules permitted payments to specialty mental health/IDD institutions, whereas such payments were prohibited for the treatment of adults.^36^ Yet further policy changes were also important. In 1971, President Nixon set a goal of reducing the number of people institutionalized with IDDs by one‐third. Congress correspondingly expanded the Social Security Amendments of 1967 to authorize Medicaid funding of care provided in Intermediate Care Facilities for the Mentally Retarded (ICF/MR) (now referred to as Intermediate Care Facilities for individuals with intellectual disabilities, ICF/IID).^35^, ^47^ States took advantage of this new funding stream to convert existing institutions for people with IDDs into ICFs, allowing them to continue providing institutional care to people with IDDs, with Medicaid matching funding.^14^ Federal policy consciously avoided the development of such arrangements for people with SPMI. States’ fiscal impetus for rapid deinstitutionalization of individuals with SPMI was therefore weaker for the IDD population, and funding for new forms of institutional care for this population grew.

In 1981, Congress created the Medicaid HCBS program, allowing states to obtain Section 1915(c) waivers to provide long‐term services in community settings to people who would otherwise qualify for institutional care.^48^ Because Medicaid covered institutional care for people with IDDs in ICFs, this population qualified for HCBS waivers. By contrast, people with SPMI generally did not qualify, because Medicaid did not cover their institutional care.^49^ Note that HCBS waivers are not an entitlement; states may cap enrollment and maintain waiting lists. Nonetheless, by 2015, only three states did not offer HCBS to at least some populations under Section 1915(c) waivers. The HCBS program does not require cost neutrality. Rather, it requires that the average cost of waiver services not exceed the cost of comparable institutional care, so the spending benchmark rises over time as institutional costs increase.

Because ICFs could receive Medicaid matching funds, oversight of these new institutions remained vested in the existing governance structure. The subsequent introduction of the HCBS program offered a full complement of Medicaid‐financed nonclinical services for persons with IDDs who live outside institutions. In the SPMI case, financing and provision of the full complement of institutional services in community settings were fragmented among provider agencies. Meanwhile, for the IDD population, Medicaid organized and financed a more complete range of services.

Between the start of deinstitutionalization and the early 1980s, the population with SPMI and that with IDDs had traveled on divergent policy paths. Medicaid, through HCBS, had begun to create an integrated system of financing and support for people with IDDs, while services for people with SPMI remained fragmented across multiple programs and agencies. We next examine how these differences affect contemporary outcomes and service systems.

## Current Status

For the large group of affected persons with SPMI and IDDs living outside of institutions, the introduction of Medicaid and SSI greatly improved conditions. These programs gave family caregivers critical resources that enabled them to care for disabled family members. They also made independent living possible for some people who would not have been able to live independently or with family before, and for whom institutionalization was the last resort. But the historical differences described earlier continue to shape the trajectories of people with SPMI and IDDs today. As discussed in the following sections, people with IDDs generally have access to a broader and more coordinated set of community supports than people with SPMI.

### Contemporary Supports and Outcomes for People With SPMI

Most people who live with SPMI have benefited from the replacement of institutional care through supports provided by Medicaid and SSI. Out of sight and out of public mind, many persons who live with mental illness have decent lives in the community, alone or with family members. They enjoy freedom and autonomy similar to those of their fellow citizens without mental illnesses. They receive health care services paid for by Medicare or Medicaid. Many have adequate access to quality care. Many whose mental illnesses cause disability have their living expenses covered or supported through SSI or Social Security Disability Income (SSDI), food stamps, and sometimes housing vouchers. Approximately 1.3 million Americans under age 65 receive SSI on the basis of psychiatric disorders. Many are socially engaged with family, clubhouses, and other social opportunities, and find some paid work.^1^

Yet a significant number fare poorly.^50^ In 2023, among the 14.6 million Americans diagnosed with a serious mental illness, about 3.5 million over the age of 18 received income support from SSI and/or SSDI.^51^ SSI income supports fail to lift many of these recipients out of poverty.^37^ About 2.31 million people with SPMI were estimated to reside in HUD‐subsidized housing.^52^, ^53^ About 13.1% of people with SPMI are employed either full or part‐time. Roughly 7% are uninsured. Finally, 2.5 million people with SPMI live on incomes below the poverty line.^54^

Many within this group, both insured and uninsured, do not receive adequate, high‐quality, continuous medical care. Many are denied income and housing supports even though their post‐denial experiences show them to be significantly impaired. Danziger, Frank, and Meara studied low‐income women whose applications for SSI were rejected.^55^ More than one‐third met diagnostic criteria for serious mental illness and more than 50% experienced housing instability at significantly higher rates than those whose applications were approved. In addition, people with diagnoses of a mood disorder (major depression or bipolar disorder) showed a denial rate of 76% compared to a denial rate for all other conditions of 62%.^56^ Some of those most seriously affected spend substantial time in jail or living on the streets, in homeless shelters, or in substandard housing. Some are viewed as threatening to themselves, to family members, or, far less often, to others.^57^, ^58^ Many experience co‐occurring substance use disorders, which have been documented as risk factors for poor outcomes and for externalizing behaviors.^59^

### Contemporary Supports and Outcomes for People With IDD

The situation of the IDD population is markedly different. Between 1982 and 2021, the estimated number of people living with IDDs who received Medicaid HCBS services grew from 1,381 to 953,571, with commensurate increases in the number receiving Medicaid‐funded long‐term services and supports (LTSS). Between 1998 and 2021, the number of persons with IDD receiving Medicaid waiver HCBS supports living in the home of a family members increased from 80,799 to 526,326 (see Larson and colleagues [2025] Figure 3.1).^46^ Over that same period, the number of LTSS recipients with IDDs living with family members receiving *non‐Medicaid* state‐funded services also markedly increased, underscoring that Medicaid and other funding streams frequently serve as complements rather than substitutes for this population. Roughly 1.8 million persons with IDDs under the age of 65 receive significant cash supports through SSI, whereas roughly 517,000 persons with intellectual disabilities (some dual‐eligible) receive income supports through the Disabled Adult Child program for surviving dependents of Social Security recipients,^60^ alongside noncash supports through Medicaid, the Supplemental Nutrition Assistance Plan, and other forms of public aid.

By 2021, 62% of people living with IDDs who received public assistance for LTSS lived with family members. Another 10% lived in their own apartments or homes.^46^ Roughly 15% lived in group home arrangements of six or fewer people. Less than 6% lived in institutional care arrangements, now defined to be any residence housing seven or more persons living with disabilities managed by a public or private service organization.^24^, ^46^ Roughly 62,000 Americans with IDDs now reside ICF/IIDs. As noted earlier, these facilities were designed to provide a less medicalized, more socially integrated and economical alternative to skilled nursing facilities and were intended to meet the specific needs of persons living with IDDs.^48^

As people with IDDs shifted from institutional to family and independent living, average annual per‐person costs for PRF residents correspondingly increased, reflecting remaining residents’ labor‐intensive service needs. In 1965, annual per‐person expenditures in PRFs were $20,305 in 2021 dollars. By 2010, this inflation‐adjusted figure had risen to $242,045. By 2021, the figure had reached $353,452, more than 17 times the 1965 value.^46^

The marshaling of impressive financial and social resources to address both medical and nonmedical needs provides another striking element in the history of IDD services. Although deinstitutionalization improved outcomes, it did not save money. In fact, real expenditures on services for people with IDDs sharply increased over the past six decades.

Daunting challenges remain in serving people with IDDs and their families. Deinstitutionalization left many formerly institutionalized individuals isolated and lonely, with unaddressed medical and service needs.^61^, ^62^ As Gettings observes: “the performance of community provider agencies remains highly uneven,” with too‐little emphasis on improving service quality within settings that are often subject to porous oversight and weak regulatory standards.^35^ One recent analysis found that among the 552,339 Americans with IDDs on waiting lists for Medicaid waiver services, 59% (329,593) resided in one state: Texas. Meanwhile, 12 states reported zero persons on their waiting list, underscoring the tremendous cross‐state variation in Medicaid policies.^49^

The high prevalence of neuropsychiatric comorbidities poses further challenges.^63^ Persons with autism disorders exhibit high rates of suicidality, depression, psychosis, and other forms of serious mental illness. These conditions are frequently overlooked through the phenomenon of “diagnostic overshadowing,” whereby clinicians mistake psychiatric challenges with symptoms of intellectual disability.^64^, ^65^ Roughly 30% of caregivers for boys and men living with Fragile X syndrome are injured by their loved one.^66^, ^67^ Public assistance and tools to assist people with IDDs can also carry punishing administrative burdens, which create intersectional disparities and compromise the ability to meet needs of all people with IDDs.^68^

Life‐cycle challenges are particularly acute. People with Down syndrome have an average life expectancy of 60 years. Yet this population faces a 95% lifetime dementia risk.^69^, ^70^, ^71^ In one nationally representative study of emergency department encounters, patients with Down syndrome between the ages of 45 and 50 displayed higher indicated dementia prevalence than found within the overall patient population over age 70.^63^, ^69^

Valuable guidelines support evidence‐informed dementia and geriatric care for persons with IDDs. These guidelines underscore the importance of co‐locating physical and mental health care, social services, housing, and vocational supports to address the intertwined needs of persons living with IDDs and those of their loved ones.^72^, ^73^ State Medicaid policymakers remain challenged to ensure that such guidelines are followed.

### Comparison of Outcomes and Expenditures Today

The differences in the structure of supports for the SPMI and IDD populations post‐deinstitutionalization translate into meaningful differences in expenditures and outcomes. These contrasts are particularly evident in a comparison of people with IDDs and those with schizophrenia. A recent study estimated that in 2021, 70% of people with schizophrenia had their health care paid for by Medicaid (a 10 percentage point increase over 2008 levels).^74^ In contrast to people with IDD, those with SPMI covered by Medicaid rarely rely on HCBS for their non‐Medicaid support for functional impairments. One recent study estimated that 2% of older adults covered by Medicaid with an SPMI made use of HCBS. Larson and colleagues note that year 2021 annual Medicaid HCBS waiver expenditures for 953,571 recipients with IDDs averaged $51,835 per waiver recipient (see Larson and colleagues Figure 3.5C).^46^ This figure itself does not include the full range of Medicaid spending for medical care. By comparison, Medicaid's expenditures to serve people with SPMI averaged roughly $21,000 per recipient that same year.^75^

Such differences are also evident in cross‐national comparisons. The US emphasis on Medicaid‐financed clinical care for the SPMI population, rather than on the broad needs that would enable them to successfully reside in the community, is evident in a comparison of the components of spending on the SPMI population in the United States and other countries. Whereas Australia and Denmark allocate about one‐third of total mental health spending on clinical care and two‐thirds on other components of care, the proportions in the United States are reversed and the outcomes are much poorer. The United States fares tangibly better in cross‐national comparisons of outcomes for persons with IDDs, though even here, gaps persist relative to other wealthy democracies.^76^, ^77^, ^78^

## Understanding the Divergence

As we showed earlier, there are substantial differences in supports and outcomes between people with IDDs and people with SPMI today. We now consider why these differences emerged. The divergent experiences of these two populations reflect both differences in the health conditions themselves and differences in the policies that evolved to support them. We consider these two sources of divergence in turn.

### Differences in Underlying Conditions and Public Perceptions of Them

Some differences in the experiences of people with SPMI and those with IDDs stem from differences in the nature of the conditions themselves. Intellectual disabilities are typically diagnosed early in life and are often fairly stable and predictable over much of an individual's life course. Parents and other caregivers can thus engage in long‐term planning regarding residential arrangements and different forms of associated care. Most adolescents and young adults with IDDs continue to live with their parents in their childhood family homes. This pattern has also fostered the emergence of persons with IDDs and their families as a key constituency that enjoyed strong support from public officials of both political parties across the United States. Such political supports provide greater opportunities for higher expenditures, and greater federal, state, and local accountability for visible service gaps or low service quality of community‐based care.

People with mental illnesses, including those with SPMI, carry stigma that is associated with perceptions of the causes of mental illnesses and the desire among many members of the public to keep their distance from this population, particularly from people who display disturbed or disturbing behavior. Research has shown that public understanding of the causes of mental illness have improved, with growing segments of the nation understanding the neurobiological underpinnings of mental illnesses. Yet a substantial plurality (31%) has consistently held the belief over decades that mental illnesses are also a consequence of a “bad character.” The desire for social distance from people with mental illnesses has remained consistent over time, with at most 52% of Americans expressing a willingness to socialize with a person with a mental illness and 35% reporting an interest in being friends with someone with a mental illness.^79^ As documented by Pescosolido and colleagues, public stigma regarding persons with major depression disorders notably declined over the period 1996–2018. Yet public stigma and the desire for physical and social distance from persons living with schizophrenia and alcohol use disorders actually increased over the same period.* ^80^ Such perceptions have colored the support for treatment of mental illnesses over time and have particularly reduced public interest in policies that could increase expenditures devoted to serving the needs of people with SPMI.

Real and perceived links between mental illness and violence has exacerbated this stigma. The availability heuristic, fueled by vivid examples of dysregulated behaviors and crimes perpetrated by people with serious mental illness, strongly influences public perceptions of proper treatment for people with SPMI and deepens local public resistance to community‐based residential services for persons who live with SPMI or other behavioral health difficulties.^81^

In fact, violence directed toward strangers is relatively uncommon among persons who experience serious mental illness. Violent behaviors directed toward intimates are far more prevalent challenges. Steadman and colleagues used data from the MacArthur Violence Risk Assessment Study to examine the prevalence of violent behaviors among 951 patients available for follow‐up after discharge from a psychiatric facility.^82^ These authors identified a total of 608 violent acts, and 262/951 patients (28%) committed at least one violent act.^82^ Among the 558 cases for which the relationship between the target and the discharged patient was known, 86% involved a family member, friend, or acquaintance. Only nine patients out of 951 perpetrated a firearm‐involved violent act or threat directed at a stranger. Swanson and colleagues reported broadly similar findings among Florida adults living with SPMI.^40^

**Figure 1:**
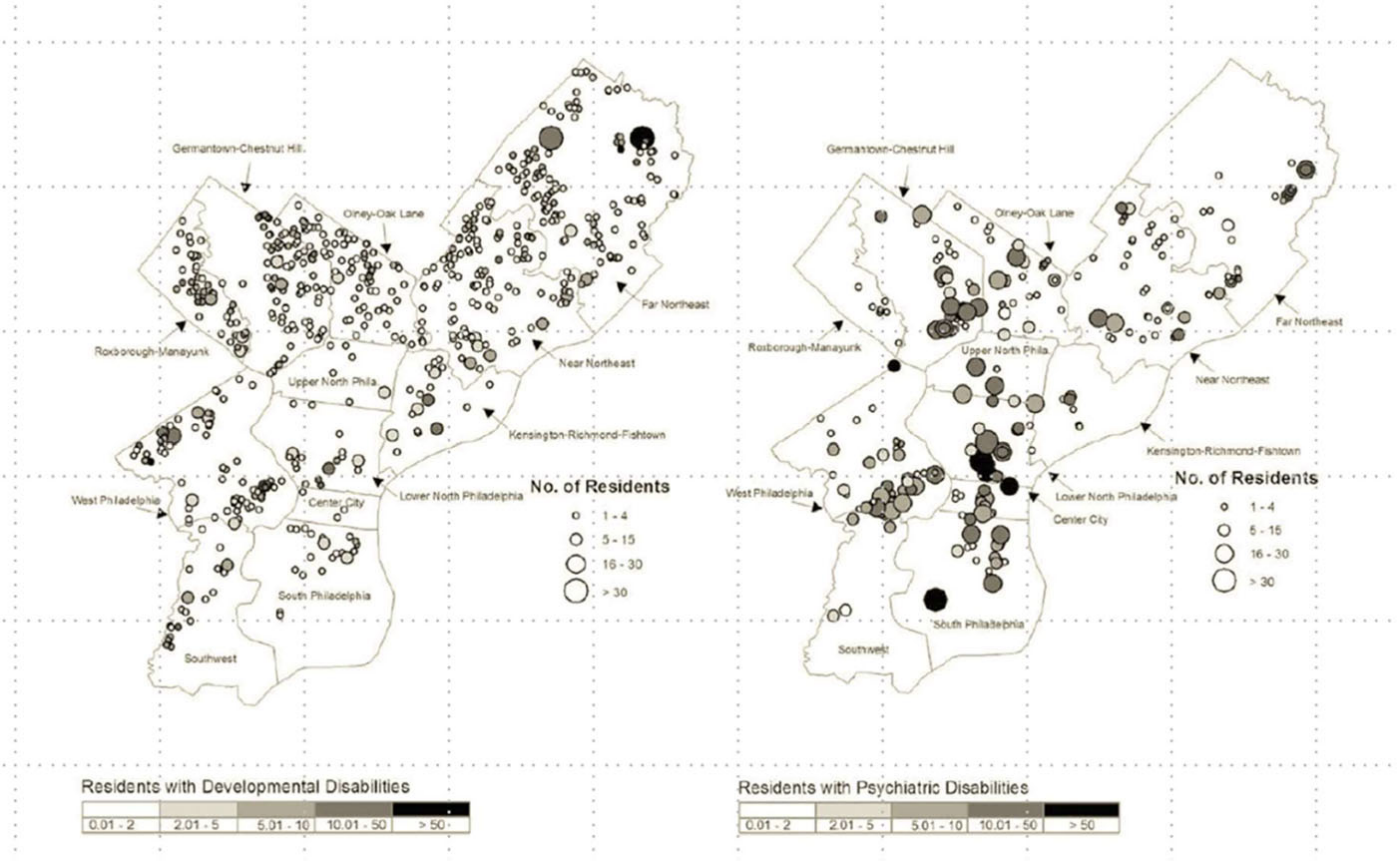
Siting of Philadelphia residential facilities for persons with IDDs and for persons with behavioral health disorders (From Wong and Stanhope 2009). [Colour figure can be viewed at wileyonlinelibrary.com]

Greater political and social acceptance of people with IDDs also plays a key role in the siting of disability services and in service delivery. Wong and Stanhope provide a striking analysis of the contrasts in social acceptance of people with IDDs and social acceptance of people living with behavioral health disorders.^83^ These authors present a geocoded map of Philadelphia that shows the size and location of residential facilities for people with the two categories of disorder. As shown in Figure 1, group homes for persons with IDD were in every city neighborhood regardless of racial composition or income.

Wong and Stanhope conclude: “the [IDD] population in supportive housing was more spatially dispersed, and lived in less distressed, less unstable, more secure, but equally racially/ethnically diverse neighborhoods when compared to the population [of persons with behavioral health disorders] in supportive housing.”

Two 2026 survey experiments by Biondi‐Bonetti, Taylor, Ma, and Pollack showed similar contrasts in local resistance to the siting of residential facilities.^84^ Within a nationally representative survey of 1,047 respondents in NORC's AMERISPEAK panel, participants were 35 percentage points more likely to support local siting of a residential facility for persons living with IDDs than they were to support local siting of a residential facility for persons living with serious mental illnesses (66.1% vs. 31.0%). These authors found extremely similar contrasts in a parallel experiment regarding local siting of a residential facility for persons living with IDD vs. those with addiction disorders (n = 1,070; 69.9% vs. 32.8%).^84^ In both experiments, roughly one‐third of respondents identified community safety as a key contrast and consideration (33.9% vs. 9.1% in the IDD vs. SPMI experiment, 29.2% vs. 5.2% in the IDD vs. addiction experiment, respectively).

Together these finding suggest that the new policy changes to Medicaid enacted by passage of H.R. 1 in 2025, more commonly known as the One Big Beautiful Bill Act, will likely have a greater effect on people with SPMI, in part, because the lines of disability can be more easily drawn for people with IDDs. Greater public sympathy for people with IDDs, along with the greater stigma and fears associated with SPMI, reinforce the functional and predictability differences. Thus one might well expect exemptions from work requirements to be more likely to protect the IDD population than those with SPMI.

### Differences in Policy Design and System Organization

The characteristics of the SPMI and IDD populations explain only part of the vast difference in outcomes. Although the 1977 GAO report hardly distinguishes between the two populations, subsequent divergences in policy have mattered a great deal. People with IDDs benefit from a structure of financing and governance that is far more coordinated and unified than the structures available to serve people with SPMI.

For the IDD population, the Medicaid HCBS program provides the bedrock over which other services are layered. No comparable foundation exists for the SPMI population.^85^ Instead, persons with SPMI are served by a set of disparate, uncoordinated programs with varied accountability and program coverage. These programs, including Medicaid and publicly supported mental health programs (e.g, CCBHCs), do not consistently prioritize the needs and consequences that accompany the most severe forms of mental illness.

## How These Differences Affect Policy Today

The outcomes of recent federal initiatives illustrate the practical consequences of this divergence between an integrated and fragmented system. We use two examples—the 988 crisis system and Medicaid waiver authorities—to show how fragmentation complicates implementation and limits accountability.

### The 988 Crisis System as a Case Study in Fragmentation

Consider the introduction of the nationwide 988 crisis line. Although police response to behavioral health crises has markedly improved through crisis intervention training and other efforts such as multidisciplinary response models for adults,^3^, ^4^, ^5^ children, and youth,^86^ violent encounters between officers and people with SPMI remain a serious challenge.^6^, ^7^ Data from December 2022 show the presence of mental illness or substance use disorder in 21% of police‐involved fatalities,^8^ while the prevalence of SPMI is at most 5% of the general population.

Mindful of such events. many family members and service providers for people with SPMI are reluctant to call 911, even when a person with SPMI exhibits behaviors that pose safety challenges to themselves or others. Murray and colleagues interviewed Chicago social service providers about their use of 911 crisis response.^6^ Most respondents noted that they would only call 911 as a last resort. Many expressed concerns about the use of force by police and the absence of therapeutic elements to the crisis interventions stemming from 911 calls.

Recognizing the desire to inject more therapeutic skills into crisis responses, the federal government and many state governments have been investing in crisis infrastructure. A key step in that effort has been newly deployed 988 systems to provide phone and text supports for people in crisis. These systems were designed to support people experiencing severe mental health crises who were considering self‐harm or harm to others and other disturbed behaviors with connection to clinical services such as mobile crisis teams.

Recent evaluations highlight the value of multidisciplinary mental health crisis response and nonpolice clinical responders as evidence‐informed approaches to crisis response to address such challenges. A striking benefit of such models is that family members are more willing to call 911 and other sources of help when mental health crises occur, secure in the knowledge that their loved one living with SPMI, IDDs, or other challenges will be provided a noncoercive and clinically informed response.^3^, ^4^ States and localities can build upon these experiences to implement scalable and sustainable crisis response models.

In practice, emergency crisis lines are serving a much broader set of problems than was originally envisioned for reasons we might expect, given the history of limited access to care. First, much of the use of the lines has been dedicated to people whose challenges do not pose significant risks to self or others. Instead, most calls involve issues that require only phone or text supports. Recent data from 988 show that the majority of callers have their problems addressed through a single call with no referral or other intervention. Although these interactions are understandable and useful, they do not reflect the goals that motivated the investments in developing crisis systems and offer a very expensive way to expand access to services that address less severe problems.

Yet because the 988 systems constitute a wholly new infrastructure, they are often disconnected from existing mental health care systems. They possess far less capacity than their 911 counterparts to provide effective linkages to immediate or follow‐on services for people in more serious difficulty, or to mobilize in‐person crisis response. In one recent study, the majority of people surveyed who called 988 would not call again.^20^ Other recent reports indicate that individuals and families who require in‐person crisis response are most likely to draw upon 911 and mental health providers already familiar to them, who have provided ongoing services and care.

The recent experience with 988 and the crisis system is reflective of long‐standing tensions in mental health policy. That is, significant public investments are motivated by the goal of addressing vexing problems associated with SPMI. Yet in execution of the policy, the populations served extend far beyond those with SPMI, and the missions of the organizations involved drift toward a broader set of mental health and social challenges.

The experience of 988 illustrates a recurring pattern in mental health policy. Because the support system for this population is so fragmented, programs intended to address particular aspects of these needs often become disconnected from the broader systems of care required to support them.

### Section 1115 and 1915 Waivers as Paths to Improvement

Medicaid policy provides a second illustration of how policy choices affect the divergent IDD and SPMI experiences. As we discussed earlier, the Medicaid HCBS waiver program created a durable platform for financing community supports for people with IDDs. By contrast, comparable waiver authorities for people with SPMI are more limited and fragmented.

Policymakers have also begun to confront the reality that people with SPMI experience a variety of health‐related social needs in domains that range from housing and transportation to nutrition supports. The Biden administration sought to address such issues by granting states Section 1115(b) Medicaid waivers that allow states to deploy a modest fraction of the nation's $918 billion combined state‐federal Medicaid expenditures to addressing health‐related social needs.^87^

While Section 1115(b) waivers allow for delivery of valuable nonclinical services for people with SPMI, the structure is inherently limited and cannot be used to establish a comprehensive HCBS‐like program. This stands in stark contrast to the budgetary requirements that govern Medicaid Section 1915(c) waivers that pursue similar goals. Recall that services provided through the HCBS 1915(c) program need only cost no more than comparable institutional services. Under the Section 1115 mechanism, investments in social determinants in principle may only be made if they are offset by reductions in Medicaid spending on traditional clinical services. The evidence for such cost offsets is very weak, limiting the potential scope of the program, particularly when many of the most valuable services to improve personal well‐being do not bring accompanying cost offsets. In other words, unlike the 1915(c) waiver, the 1115 mechanism does not conceive of the need for nonclinical services for people with SPMI as being in scope for Medicaid‐funded services.

Such contrasts underscore the fundamental differences between the Medicaid experiences of people with IDDs and those with SPMI. Since 1981, the Medicaid program has served as a unifying focus for nearly the full range of services needed to successfully support people with IDDs. In contrast, requisite services and supports for people with SPMI have been far more fragmented. Medicaid finances clinical and some support services. Much of the nonclinical and human services for people with SPMI are spread across an array of federal, state, and local public agencies, with a range of differing and complex eligibility standards, rules, and regulations.

## Policy Lessons for Strengthening Community‐Based Care

The federal government must provide states with authorities under Medicaid that allow greater use of HCBS for people with SPMI. Concretely, that means deemphasizing authorities that require budget neutrality in a post‐institutionalization world (Section 1115 waivers) and instead relying on those with greater budget flexibility (Section 1915 waivers).

The expertise of state mental health authorities must also be incorporated into oversight and quality improvement efforts in Medicaid. The federal government, in its design of new mental health programming, must establish regulations and oversight that permit states to effectively pursue policy intents. This includes establishing federal rules for obtaining grants for new capacity (e.g., CCBHCs) that are sufficiently flexible to recognize the diversity of communities involved in supporting people with SPMI. Finally, when federal programs that intend to deal with consequences of severe mental illnesses are provided through broad authorities (e.g., 988 call lines), states must take greater care to create expectations and lines of accountability that align with the federal intent of the program and limit tendencies toward mission drift.

Our comparison reveals a simple lesson. People with IDDs and people with SPMI both benefited from deinstitutionalization, but only the IDD population subsequently gained access to a relatively coherent and accountable system of community supports.

Looking ahead, states face many challenges in strengthening their specific focus to meet the full spectrum of needs experienced by the most vulnerable Americans who live with SPMI. Accomplishing this task requires states to enrich the governance structures deployed to meet these needs and to build sustainable and capable infrastructures to ensure that services are available to those who need them and are delivered well.

State policymakers, their federal colleagues, and the American public must also embrace key lessons from our relatively more successful national commitment to meet the needs of Americans with IDDs and their families. Our first obligation is to meet human needs of affected persons, families, and communities, many of whom are now underserved within the arena of clinical care but also across a range of economic and social needs. Effective policies to meet these needs will not save money. They will, however, save and improve millions of lives.

## Funding/Support

None to disclose

## Conflict of Interest Disclosures

None to disclose.

## Footnotes

* We thank Breanne Biondi for drawing our attention to this reference.
